# Transcatheter tricuspid valve intervention versus medical therapy for symptomatic tricuspid regurgitation: a meta-analysis of reconstructed time-to-event data

**DOI:** 10.1097/JS9.0000000000001773

**Published:** 2024-06-13

**Authors:** Guangguo Fu, Jianfu Zhu, Wenyu Song, Ghufran Bagaber, Chunsheng Wang, Jinmiao Chen, Lai Wei

**Affiliations:** a Department of Cardiovascular Surgery, Zhongshan Hospital, Fudan University, Shanghai, China; b Shanghai Institute of Cardiovascular Diseases, Zhongshan Hospital, Fudan University, Shanghai, China; c Shanghai Medical College, Fudan University, Shanghai, China; d Department of Cardiovascular Surgery, Shanghai Geriatric Medical Center, Shanghai, China

**Keywords:** medical therapy, meta-analysis, transcatheter tricuspid valve intervention, tricuspid regurgitation

## Abstract

**Background:**

Transcatheter tricuspid valve intervention (TTVI) has demonstrated safety and efficacy in treating high-risk patients with tricuspid regurgitation (TR). The authors aimed to perform a meta-analysis based on reconstructed time-to-event data to compare the clinical benefit of TTVI with medical therapy (MED).

**Methods:**

A systematic literature search was conducted in major databases, including PubMed, Embase, and the Cochrane Library, until 20 October 2023. All studies comparing the outcomes between TTVI and MED were included. The primary outcome was all-cause mortality. The secondary outcomes included heart failure (HF) hospitalization and the composite outcome of all-cause mortality and HF hospitalization.

**Results:**

Five studies covering 3826 patients (1146 received TTVI and 2680 received MED) were identified. At 1-year follow-up, TTVI significantly reduced the risk of all-cause mortality compared with MED [hazard ratio (HR) 0.54, 95% CI: 0.39–0.74, *P*=0.0001]. There was a trend in favor of TTVI in HF hospitalization, although without significant difference (HR 0.70, 95% CI: 0.42–1.18, *P*=0.18). TTVI was also associated with a decreased risk of composite outcome (HR 0.57, 95% CI: 0.38–0.86, *P*=0.007). Reconstructed Kaplan–Meier curves illustrated a 1-year overall survival rate of 83.1% in the TTVI group and 68.8% in the MED group. The subgroup analysis of device types yielded consistent results.

**Conclusions:**

Compared with MED, TTVI was associated with greater 1-year benefits for patients with symptomatic moderate or greater TR from the aspects of all-cause mortality and HF hospitalization.

## Introduction

Highlights

- The first meta-analysis comparing transcatheter tricuspid valve intervention with medical therapy.
- Transcatheter tricuspid valve intervention provided greater survival benefits compared with medical therapy.
- This meta-analysis was based on the reconstructed time-to-event data.

Tricuspid regurgitation (TR), a common but underrecognized condition, accounts for the vast majority of tricuspid valve (TV) diseases. The overall prevalence of moderate or severe TR is 0.55% and is strongly related to age^1^. It is estimated that 1 in 25 people over the age of 75 years suffers from moderate or severe TR^1^. Thus, the number of patients with TR is expected to grow even further with the accelerated aging of the population^2^.

It has been recognized that significant TR adversely impacts cardiac mortality and heart failure (HF) hospitalization^3–6^. However, the medical therapy (MED) for TR is limited to medications for HF, with diuretics as the primary treatment for palliative care^7,8^. As for surgical treatment, less than 4% of patients with functional TR undergo isolated TV surgery after diagnosis, and the perioperative mortality rate of isolated TV surgery remains as high as 8–10%^4,9–12^. Currently, due to the relatively small number of isolated TV surgery, there is no widespread recommendation for isolated TV surgery in either the ACC/AHA guidelines or the ESC/EACTS guidelines^7,13^. There is still a lack of sufficient long-term evidence from randomized clinical trials (RCTs) to support the actual benefit of isolated TV surgery. Although minimally invasive surgery may reduce the surgical mortality, the surgical volume remains low^11,12,14–17^.

The emergence and development of transcatheter tricuspid valve intervention (TTVI) has provided new choices for patients with TR at high surgical risk. Several single-arm trials and real-world studies have reported a lower periprocedural mortality rate of 1–3% for transcatheter intervention^18–21^. Although there have been several observational studies demonstrating the greater efficacy of TTVI over MED^22,23^, RCTs with solid evidence comparing TTVI and MED in large samples are still scarce. Therefore, we conducted a comprehensive meta-analysis with reconstructed time-to-event data from RCT and observational studies to quantitatively assess the efficacy of TTVI and MED.

## Materials and methods

The original protocol of this meta-analysis has been registered in the International Prospective Register of Systematic Reviews (PROSPERO) and was conducted in accordance with the Preferred Reporting Items for Systematic reviews and Meta-Analyses (PRISMA, Supplemental Digital Content 2, http://links.lww.com/JS9/C754, Supplemental Digital Content 3, http://links.lww.com/JS9/C755) guideline^24^ and Assessing the Methodological Quality of Systematic Reviews 2 (AMSTAR 2, Supplemental Digital Content 4, http://links.lww.com/JS9/C756) guidelines^25^. Given the nature of our work, ethical approval was not required for this study.

### Search strategy

We performed a systematic search in the major databases including PubMed, Embase, and the Cochrane Library, until 20 October 2023. The following keywords and medical subject heading (MeSH) terms were used for the search: (Transcatheter OR Percutaneous OR Catheterization) AND (Intervention OR Repair OR Replacement OR Implantation) AND (Medical Therapy OR Drug Therapy OR Conservative Treatment) AND (Tricuspid Valve Regurgitation OR Tricuspid Valve Insufficiency). The detailed search strings were available in Supplementary Materials (Supplemental Digital Content 1, http://links.lww.com/JS9/C753). Furthermore, the reference lists of all included articles, relevant reviews, and meta-analyses were also carefully screened to identify potentially eligible studies.

### Study selection

After the removal of duplicates, two authors (G.F. and J.Z.) independently screened the literatures following the order of title, abstract, and full text. Any discrepancies during the screening process were resolved through discussion and consensus. Our inclusion criteria was as follows: (1) studies comparing TTVI with MED in the patients with TR; (2) studies presenting at least one of the following results or Kaplan–Meier curves: all-cause mortality, HF hospitalization, or the composite outcome of all-cause mortality and HF hospitalization; (3) studies written in English. No restriction on the study design was applied. Conference abstracts, reviews, meta-analyses, first-in-human experiences, case reports, editorials, and letters were all excluded. If two distinct studies were conducted on populations that might overlap, the study with a smaller sample size would be excluded unless it provided an outcome or Kaplan–Meier curve that was not available in the other study.

### Quality assessment and data extraction

Two authors (G.F. and J.Z.) independently assessed the quality of the included studies and extracted the data from the included studies. When there was any disagreement, a third review author (J.C.) validated the data and resolved the disagreement to reach consensus. The included RCTs were assessed using the Cochrane Risk of Bias 2 (RoB 2) tool^26^ and the included retrospective studies were assessed using the Cochrane Risk of Bias in Non-Randomized Studies of Interventions (ROBSIN-I) tool^27^.

The data were extracted regarding study characteristics (number of patients in each group, country or region, number of centers, study period, follow-up duration, study design, severity of TR and device), patient baseline characteristics [age, female, BMI, atrial fibrillation, diabetes mellitus, chronic kidney disease, functional TR, TR ≥severe, New York Heart Association (NYHA) class ≥III, and tricuspid annular plane systolic excursion (TAPSE)] and the clinical outcomes as mentioned above.

### Endpoints and definitions

The primary outcome was all-cause mortality, including any cardiovascular mortality and noncardiovascular mortality. The secondary outcomes included HF hospitalization and the composite outcome of all-cause mortality and HF hospitalization. If the composite outcome was not directly reported in the included literatures, HF hospitalization was preferred, followed by all-cause mortality, as an approximation to substitute for the composite outcome.

### Statistical analysis

The individual patient data (IPD) were extracted as reconstructed time-to-event data from any available Kaplan–Meier curves for primary or secondary outcomes provided by the included studies. We obtained IPD using the R package ‘IPDfromKM’ (version 0.1.10) following the standard two-step method^28^. First, we extracted the raw data coordinates including time and corresponding survival probability from each point on each group of curves in the Kaplan–Meier plots. Second, we collected the time points and the number of patients at risk from the risk tables, and then combined the time points and the number at risk with the raw data coordinates to reconstruct IPD including follow-up time for each patient and corresponding event status.

When IPD were obtained, we utilized the Cox proportional hazards model to calculate the estimated hazard ratios (HRs) and 95% CIs for individual Kaplan–Meier curves through the R package ‘survival’ (version 3.5.7). If a study directly reported the HR and 95% CI value calculated from its original data, we prioritized that reported value and discarded our corresponding estimated HR value. Then the directly reported values and the retained IPD-derived values of HR with 95% CI were pooled to calculate the estimated overall effects for survival analysis. The natural logarithms of HR and corresponding standard error were calculated, and random effects models with the Inverse-Variance method were chosen to evaluate the overall HRs and 95% CI. Similarly, we also gathered the number of events and total patients from included studies to calculate the overall risk ratio (RR) and 95% CI. The results of pooled outcomes were visualized in forest plots and the heterogeneity among different studies was investigated by the *χ*² test and quantified by the *I* ^2^ statistic.

Additionally, we merged IPD and then fitted the reconstructed Kaplan–Meier curves using the R package ‘survminer’ (version 0.4.9). The Cox proportional hazards model was also utilized to calculate the overall HRs and 95% CIs for merged IPD. Flexible parametric survival models such as Royston-Parmar models or generalized survival models were adopted to reveal the trend of HR values over time^29^. To compare the lifetime lost, we assessed the restricted mean survival time (RMST) through the R package ‘survRM2’ (version 1.0.4)^30^.

Potential publication bias was shown in funnel plots and evaluated statistically by Begg’s test, Egger’s test, and arcsine-Thompson’s test. To test the robustness of results and to evaluate whether the results were primarily dominated by single studies, sensitivity analyses were conducted using study-level leave-one-out analyses. In addition, subgroup analyses were explored based on device and study design.

The pooled survival analyses of estimated overall effects were conducted using Review Manager (version 5.4.1, The Cochrane Collaboration, 2020) and the other analyses were conducted using R Statistical Software (version 4.3.2, Foundation for Statistical Computing in Vienna, Austria). *P*-values less than 0.05 were considered statistically different.

## Results

### Study characteristics

Overall, a total of 2206 records were identified through initial database searching. According to our inclusion criteria, five studies^22,23,31–33^ were finally included (Supplementary Figure S1, Supplemental Digital Content 5, http://links.lww.com/JS9/C757). Among them, one study was designed as an RCT, and the others were retrospective studies. These studies covered 3826 patients, of whom 1146 underwent TTVI and 2680 underwent MED. The main characteristics of the included studies were presented in Table 1 and the baseline characteristics of the study population were summarized in Table 2. The quality assessment results based on the Cochrane tool indicated an overall low risk of bias and high quality of all included studies (Supplementary Figure S2, Supplemental Digital Content 5, http://links.lww.com/JS9/C757).

**Table 1:** Main characteristics of the included studies.

Study	Patients (n) TTVI / MED	Country or Region	Number of centers	Study period	Follow-up duration (mo)	Study design	Severity of tricuspid regurgitation	Device
Sorajja 2023	175 / 175	Europe and North America	65 (TRILUMINATE pivotal trial)	2019.8.21–2021.9.29	12	RCT	≥Severe	TriClip
Scotti 2023	556 / 2072	Europe and North America	TTVI 24 (TriValve registry); MED 1	TTVI 2016–2021 MED 2015–2018	12	IPTW	TTVI ≥Moderate; MED ≥Severe	Any available TTVI^a^
Kresoja 2020	94 / 94	Germany	TTVI 2; MED 1	2016.7–2019.4	7.9 (5.8–12.2)^b^	PSM	≥Moderate to severe	MitraClip
Cai 2020	53 / 71	Canada	1	2015.12.1–2019.9.1	TTVI 14 (7.0–25.0)^b^ MED 17 (9.5–23.0)^b^	Unmatched	≥Severe	MitraClip
Taramasso 2019	268 / 268	Europe and North America	TTVI 22 (TriValve registry); MED 2	2016-2018	12	PSM	TTVI ≥Severe; MED ≥Moderate	Any available TTVI^a^

^a^ Includes any available transcatheter repair and replacement devices.

^b^ Median (25th–75th percentiles).

IPTW, inverse probability of treatment weighting; MED, medical therapy; NA, not available; PSM, propensity score matching; RCT, randomized controlled trial; TTVI, transcatheter tricuspid valve intervention

**Table 2:** Baseline characteristics of patients from the included studies.

Study	TTVI versus MED										
	Patients, *n*	Age, years	Female, %	Body mass index, kg/m^2^	Atrial fibrillation, %	Diabetes mellitus, %	Chronic kidney disease, %	Functional tricuspid regurgitation, %	Tricuspid regurgitation ≥ severe, %	NYHA class ≥ III,%	TAPSE, mm
Sorajja 2023	175 / 175	78.0 (7.4) 77.8 (7.2)	56.0 / 53.7	27.0 (5.8) 26.9 (5.2)	87.4 / 92.6	16.0 / 15.4	35.4 / 35.4	94.8 / 92.9	97.7 / 98.8	59.4 / 55.4	48.0 / 48.0^a^
Scotti 2023 Women cohort	316 / 1335	74.3 (11.6) 74.6 (15.1)	100 / 100	26.5 (5.8) 28.9 (9.0)	43.2 / 40.1	41.6 / 35.6	52.0 / 50.7	88.8 / NA^b^	NA / NA	92.7 / NA^b^	16.6 (5.0) 17.3 (5.1)
Scotti 2023 Men cohort	240 / 737	74.5 (9.3) 71.4 (15.0)	0 / 0	26.6 (4.9) 28.9 (6.9)	59.6 / 47.5	36.3 / 30.1	53.6 / 53.3	88.8 / NA^b^	NA / NA	92.7 / NA^b^	16.7 (4.7) 18.3 (6.0)
Kresoja 2020 HFrEF cohort	33 / 33	77.0 (72.6–81.0) 77.0 (67.5–82.5)	49 / 46	26.0 (23.5–29.9) 25.8 (22.8–26.2)	82 / 70	NA / NA	55 / 49	100 / 100	100 / 97	85 / 85	15 (5) 16 (4)
Kresoja 2020 HFpEF cohort	61 / 61	77.3 (73.6–81.0) 78.0 (73.0–82.5)	53 / 64	25.2 (23.0–30.3) 25.1 (22.0–27.8)	92 / 79	NA / NA	33 / 41	100 / 100	93 / 94	85 / 64	17 (5) 18 (5)
Cai 2020	53 / 71	74.8 (11.1) 77.2 (9.6)	58.5 / 42.3	25.5 (6.3) 24.7 (5.0)	88.7 / 90.1	30.2 / 29.6	37.8 / 19.7	88.7 / 93.0	100 / 100	93.5 / 67.6	16.3 (4.5) 16.3 (4.5)
Taramasso 2019	268 / 268	77 (8) 76 (13)	56 / 59	NA / NA	82 / 50	NA / NA	NA / NA	90 / 95	NA / NA	93 / 23	NA / NA

Continuous variables were presented as the mean (SD) or median (25th–75th percentiles). Categorical variables were presented as percentages.

^a^ The percentage of patients with TAPSE value ≥17 mm was 48.0% in both the TTVI and MED groups.

^b^ This article only provided the overall percentages in the TTVI group (women cohort plus men cohort).

HFpEF, heart failure with preserved ejection fraction; HFrEF, heart failure with reduced ejection fraction; MED, medical therapy; NA, not available; NYHA, New York Heart Association; TAPSE, tricuspid annular plane systolic excursion; TTVI, transcatheter tricuspid valve intervention.

### Primary outcome

#### All-cause mortality

**Figure 1:**
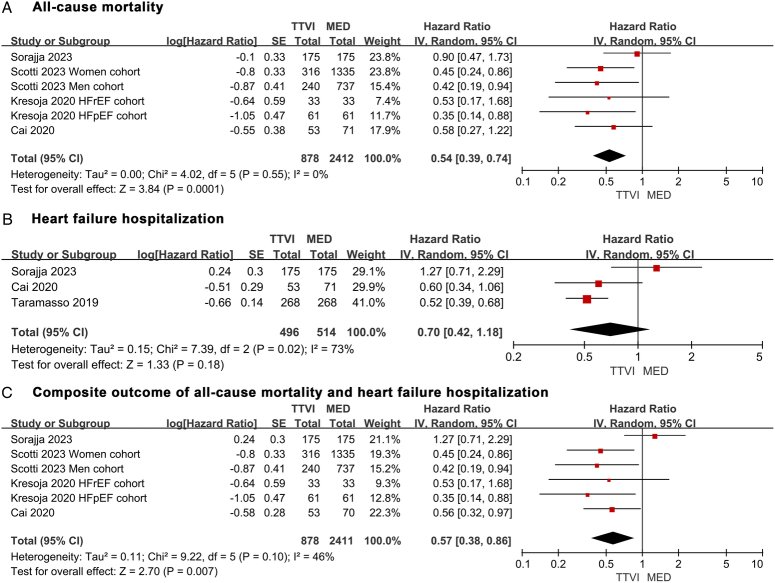
Forest plots showing the pooled 1-year outcomes for hazard ratio. (A) All-cause mortality. (B) Heart failure hospitalization. (C) The composite outcome of all-cause mortality and heart failure hospitalization. IV, inverse-variance; MED, medical therapy; TTVI, transcatheter tricuspid valve intervention.

**Figure 2:**
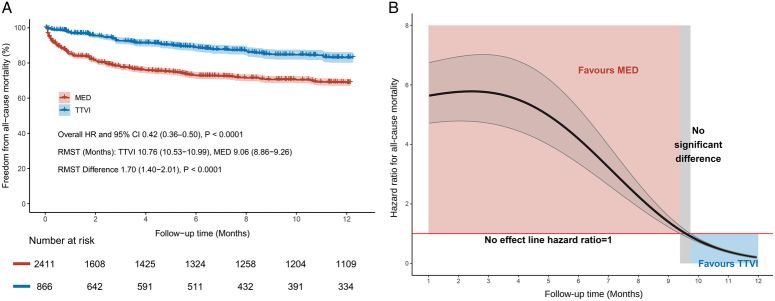
(A) Reconstructed Kaplan–Meier curves of pooled individual patient data for all-cause mortality in TTVI and MED. (B) The trend of hazard ratio over time for all-cause mortality with TTVI versus MED. CI, confidence interval; HR, hazard ratio; MED, medical therapy; RMST, restricted mean survival time; TTVI, transcatheter tricuspid valve intervention.

A total of four studies included in the analysis of all-cause mortality covered 3290 patients (878 received TTVI vs. 2412 received MED). At 1-year follow-up, TTVI significantly reduced the risk of all-cause mortality compared with MED (HR 0.54, 95% CI: 0.39–0.74, *P*=0.0001). No statistical heterogeneity was detected (*I* ^2^=0%) (Fig. 1A). The reconstructed Kaplan–Meier curves based on the four studies clearly illustrated a considerable difference in overall survival rate between the two groups at 1-year (TTVI 83.1% vs. MED 68.8%). The results obtained through merged IPD also suggested a significantly lower risk of all-cause mortality for the TTVI group (overall HR 0.42, 95% CI: 0.36–0.50, *P*<0.0001). The RMST was overall 1.70 months significantly longer in the TTVI group than the MED group (10.76 months vs. 9.06 months, *P*<0.0001) (Fig. 2A). The trend of HR values over time for all-cause mortality demonstrated that survival benefit favored the MED group during the first 9.38 months. However, the survival advantage of MED turned into a significant disadvantage compared with TTVI after 9.74 months (Fig. 2B). The subgroup analysis of patients in different types of devices yielded consistent results, regardless of TriClip and MitraClip (HR 0.62, 95% CI: 0.41–0.93, *P*=0.02), or any available TTVI devices (HR 0.44, 95% CI: 0.26–0.72, *P*=0.001) (Supplementary Figure S3A, Supplemental Digital Content 5, http://links.lww.com/JS9/C757). The subgroup analysis of different study designs revealed a trend toward a higher survival benefit without statistical difference for TTVI in the included RCT (HR 0.90, 95% CI: 0.47–1.73, *P*=0.76). In contrast, the pooled results of the other non-RCT studies remained significantly favorable for TTVI (HR 0.46, 95% CI: 0.32–0.66, *P*<0.0001). There was moderate heterogeneity between the subgroups (*I* ^2^=68.9%) (Supplementary Figure S3B, Supplemental Digital Content 5, http://links.lww.com/JS9/C757).

### Secondary outcomes

#### HF hospitalization

**Figure 3:**
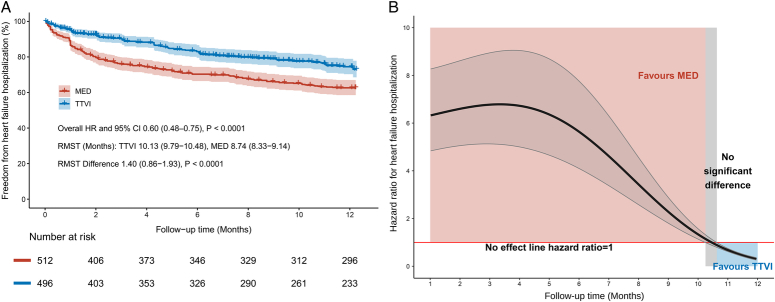
(A) Reconstructed Kaplan–Meier curves of pooled individual patient data for heart failure hospitalization in TTVI and MED. (B) The trend of hazard ratio over time for heart failure hospitalization with TTVI versus MED. CI, confidence interval; HR, hazard ratio; MED, medical therapy; RMST, restricted mean survival time; TTVI, transcatheter tricuspid valve intervention.

A total of three studies included in the analysis of HF hospitalization covered 1014 patients (496 received TTVI vs. 514 received MED). At 1-year follow-up, there was a trend in favor of TTVI, although no significant difference was observed (HR 0.70, 95% CI: 0.42–1.18, *P*=0.18). There was moderate statistical heterogeneity (*I* ^2^=73%) (Fig. 1B). The Kaplan–Meier curves reconstructed from the three studies demonstrated a considerable difference in the overall rate of freedom from HF hospitalization between the two groups at 1-year (TTVI 73.0% vs. MED 62.6%). The results from merged IPD similarly indicated a significantly lower risk of HF hospitalization for the TTVI group (overall HR 0.60, 95% CI: 0.48–0.75, *P*<0.0001). The RMST was overall 1.40 months significantly longer in the TTVI group than the MED group (10.13 months vs. 8.74 months, *P*<0.0001) (Fig. 3A). The trend of HR values over time showed that the benefit of freedom from HF hospitalization favored the MED group during the first 10.20 months. Nevertheless, the benefit turned from favoring MED to favoring TTVI after 10.63 months (Fig. 3B).

### Composite outcome

**Figure 4:**
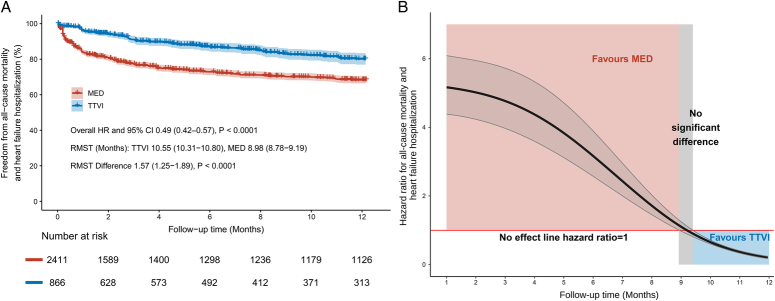
(A) Reconstructed Kaplan–Meier curves of pooled individual patient data for the composite outcome of all-cause mortality and heart failure hospitalization in TTVI and MED. (B) The trend of hazard ratio over time for the composite outcome of all-cause mortality and heart failure hospitalization with TTVI versus MED. HR, hazard ratio; MED, medical therapy; RMST, restricted mean survival time; TTVI, transcatheter tricuspid valve intervention.

A total of four studies included in the analysis of the composite outcome covered 3289 patients (878 received TTVI vs. 2411 received MED). At 1-year follow-up, TTVI significantly decreased the risk of composite outcome compared with MED (HR 0.57, 95% CI: 0.38–0.86, *P*=0.007). Mild statistical heterogeneity was observed (*I* ^2^=46%) (Fig. 1C). The Kaplan–Meier curves aggregated from the pooled four studies revealed a great difference in the overall rate of freedom from all-cause mortality and freedom from HF hospitalization between the two groups at 1-year (TTVI 80.3 vs. MED 68.3%). The results from the merged IPD also suggested a significantly lower risk of HF hospitalization for the TTVI group (overall HR 0.49, 95% CI: 0.42–0.57, *P*<0.0001). The RMST was overall 1.57 months significantly greater in the TTVI group than the MED group (10.55 months vs. 8.98 months, *P*<0.0001) (Fig. 4A). The trend of HR values for all-cause mortality and HF hospitalization showed that MED provided more survival benefits during the first 8.91 months, while the survival benefit was subsequently changed to be contributed by TTVI after 9.40 months of follow-up (Fig. 4B).

### Publication bias and sensitivity analysis

The funnel plots of all-cause mortality, HF hospitalization, and the composite outcome were depicted in Supplementary Figure S4 (Supplemental Digital Content 5, http://links.lww.com/JS9/C757). Visual inspection of the funnel plots and the results of Begg’s test, Egger’s test, and arcsine-Thompson’s test suggested that there was no significant publication bias (Supplementary Table S1, Supplemental Digital Content 5, http://links.lww.com/JS9/C757). The consistent results in the forest plot of overall RR for all outcomes (Supplementary Figure S5, Supplemental Digital Content 5, http://links.lww.com/JS9/C757) and in the leave-one-out analysis for all-cause mortality (Supplementary Table S2, Supplemental Digital Content 5, http://links.lww.com/JS9/C757) confirmed the robustness of our analyses.

## Discussion

The present meta-analysis demonstrated greater 1-year benefits of TTVI compared with MED for all-cause mortality, HF hospitalization, and the composite outcome of all-cause mortality and HF hospitalization, in patients with symptomatic moderate or greater TR. Irrespective of the device type, TTVI was associated with improvements in all-cause mortality. However, different types of study design showed heterogeneous outcomes.

To our knowledge, this is the first meta-analysis that compared the clinical outcomes of patients who received TTVI versus those who received MED alone. Several meta-analyses^34–38^ have previously reported the clinical efficacy in high-risk TR patients who underwent transcatheter repair, from the aspects of NYHA class, 6 min walk distance, the severity of TR, TAPSE, and systolic pulmonary artery pressure. Improvements in cardiac function and echocardiographic parameters after transcatheter repair proved to be statistically significant in these meta-analyses. However, these analyses based on single-arm observational studies were only able to compare the prerepair and postrepair status of the patients themselves. The absence of a control arm rendered these studies more prone to bias. Our study including the MED group could well address such inherent bias. The improved functional and echocardiographic indices in previous studies offered a partial explanation regarding the greater survival benefit of TTVI over MED for our findings. These consistent results strengthen the authenticity and acceptability of our study.

Probably due to the lower number of patients in the TTVI group, the invasive injuries and potential early complications associated with TTVI procedures, the trend curves of HR indicated that the risk of endpoint events in the TTVI group was not lower than in the MED group initially after procedure. Nevertheless, as the follow-up time increased, the HR values continued to decrease and the superiority of TTVI became evident at ~9 or 10 months. It could be hypothesized that longer follow-up after TTVI may be necessary to allow adequate reverse remodeling and recovery of RV function in the severely compromised right ventricles of high-risk patients. The survival analysis conducted by Cai *et al*.^22^ revealed a similarly significant difference in the survival advantage of TTVI compared with MED after 12 months and up to 30 months.

The underlying pathophysiologic mechanisms of the beneficial effects from TTVI are not yet exactly understood. The decreased regurgitant volume associated with TTVI may contribute to an increase in the RV stroke volume, a reduction in RV end-diastolic volume, and a decrease in RV wall tension^39–41^. The reduction of RV volume overload reversed RV remodeling, which interrupted the vicious cycle among TR deterioration, TV annulus expansion, and RV dysfunction^23,37,41^. In addition, improved RV function promotes blood circulation in the pulmonary vessels, which improves left ventricular filling and facilitates ventricular interactions^33,42^. The clinical outcomes for complications were not widely available in the included articles, thus we did not report the overall HR value for these outcomes. The advantage of lower incidence of gastrointestinal bleeding, acute renal failure, stroke, and so on, in the TTVI group has been presented in several studies^22,34^. This is probably owing to the alleviation of peripheral venous congestion and fluid retention through improved RV function, which ameliorates the visceral organ impairment.

While the pooled results showed an overall superiority of TTVI in the primary outcome, the heterogeneity presented in the subgroup analyses for the study design was worth discussing. As a landmark RCT in the field of TV intervention, the TRILUMINATE pivotal trial^31^ demonstrated that transcatheter tricuspid edge-to-edge repair was effective in improving valve function and quality of life in comparison with MED. Nevertheless, in contrast to the observational studies, the hard endpoints of all-cause mortality and HF hospitalization in this RCT were not detected to be significantly different. There might be several explanations for such heterogeneity. First, despite a mean age of nearly 80 years, the proportion of NYHA class ≥III was less than 60% and the 1-year survival probability was more than 90%, indicating a relatively better condition and tolerability in the enrolled patients. Second, a 1-year follow-up might not be sufficient to find a considerable difference.

In RCTs, the rigorous screening and randomization of enrolled patients provides robust results that minimize confounders. In observational studies, the results tend to reflect the actual benefit for the enrolled patients in the real-world. Hence, the appropriate selection of patients for TTVI is a key concern. Based on available data, it is still not clear which subgroup of patients with TR will get more benefit from TTVI. Therefore, it is not reasonable to consider TTVI as a one-size-fits-all treatment option. As shown in an included retrospective study, the treatment effect provided by TTVI was considered different, for patients with HF with reduced ejection fraction versus with preserved ejection fraction^33^. Another retrospective study from TriValve registry has described a U-shaped relationship between RV function and the potential benefits of TTVI compared to conservative therapy, with the greatest therapeutic benefit observed in patients with mid-range RV function^43^. In future endeavor, patients with different levels of left-heart or right-heart function should be considered important target subgroups, and data on the performance of left-heart or right-heart function in the comparison of TTVI versus other treatments are warranted. These data contribute to refining patient selection for TTVI and to our deeper understanding regarding the importance of the coherence of overall cardiac function when treated with TTVI.

The 2021 ESC/EACTS guidelines recommend transcatheter treatment for symptomatic but inoperable patients with functional severe TR at only level 2B^7^, which illustrates the underutilization of TTVI. Our findings further supported and strengthened the role of this recommendation in guiding clinical decision-making, and might contribute to the increased attention and further expansion of TTVI use. However, further large-scale RCTs or real-world studies that compare the outcomes of TTVI with MED or surgery are needed to establish TTVI as a first-line recommendation in future guidelines. The results of TRISCEND II Trial (NCT04482062), TRI-FR Trial (NCT04646811), and CLASP II TR Trial (NCT04097145) are worth awaited to yield further insights into the therapeutic efficacy of TTVI versus MED.

Furthermore, the overall healthcare costs for the target population of TTVI may increase with the aging of society, especially when the potential population has significant comorbidities and the use of transcatheter techniques is expanded and costly. In other heart valve diseases, transcatheter treatments prolong quality-adjusted life expectancy and increase costs within acceptable thresholds compared with MED^44,45^. It is critical to explore whether TTVI follows the similar cost-effectiveness pattern in future investigations. For patients, not only do we clinicians need to fully inform the patients of the potential improvement in quality of life and survival advantage, but also of the potential risk of procedural complications and expected recovery time from TTVI. We should always consider the patient’s preference during the decision-making process^46^.

The issues including the optimal timing of TR treatment, the durability of TTVI, and the comparison of transcatheter repair and replacement will likewise require resolution in the future^8,47,48^. Given the high complexity of TR, it is noteworthy that study design should adopt various novel technologies, such as multimodal imaging and artificial intelligence. These novel tools are able to integrate multiple variables (including circulating biomarkers) and analyze large datasets, which may facilitate personalized and precise interventions for patients with TR at the optimal time of treatment^49^. The durability and the comparison of different devices require more data from future trials with more advanced devices, larger samples, and longer follow-up periods.

### Limitation

We acknowledged that several limitations hinder the reliability and acceptability of our findings. First, only a maximum of four studies were pooled and analyzed in each outcome, which could raise potential publication bias. Second, the majority of the included studies were observational studies. Nonrandomization of patient assignment in observational studies might introduce selection bias and other undetectable confounders. Third, the reconstructed IPD analysis we conducted was not a true IPD meta-analysis based on actual IPD. Our analysis relied on the quality of the available Kaplan–Meier curves from included studies. Thus, we could only perform study-level rather than detailed patient-level subgroup analyses. Fourth, although meta-analyses based on the time-to-event data offer a useful tool to extract information from RCTs and observational studies, our results should be interpreted as explorative and hypothesis-generating and need more verification in large-scale prospective trials or real-world investigations.

## Conclusion

This meta-analysis demonstrated that in patients with symptomatic moderate or greater TR, TTVI was associated with greater 1-year benefits for all-cause mortality and HF hospitalization compared with MED. The survival advantage provided by TTVI was independent of device type.

## Ethical approval

No ethical approval was required in this study.

## Consent

No ethical approval was required in this study.

## Source of funding

This work was supported by the National Natural Science Foundation of China (No. 82200526), the Shanghai Rising-Star Program (No. 23QB1400900), the Shanghai ‘Rising Stars of Medical Talents’ Youth Development Program (No. SHWRS2023-62) and the Clinical Research Fund of Shanghai Municipal Health Commission (No. 20224Y0286).

## Author contribution

G.F., J.C., and L.W.: conceptualization; G.F. and J.Z.: data curation; G.F., J.Z., and G.B.: formal analysis; L.W. and J.C.: funding acquisition; G.F., J.Z., and W.S.: investigation; G.F., J.Z., and W.S.: methodology; L.W. and C.W.: project administration; L.W. and J.C.: resources; G.F. and J.Z.: software; L.W. and C.W.: supervision; L.W., J.C., and C.W.: validation; G.F., J.Z., and G.B.: visualization. All authors contributed in roles/writing – original draft and writing – review and editing.

## Conflicts of interest disclosure

All authors declare that there are no conflicts of interest.

## Research registration unique identifying number (UIN)

1. Name of the registry: Transcatheter tricuspid valve intervention versus medical therapy in patients with tricuspid regurgitation: a systematic review and meta-analysis
2. Unique identifying number or registration ID: CRD42023459942.
3. Hyperlink to your specific registration (must be publicly accessible and will be checked): https://www.crd.york.ac.uk/PROSPERO/display_record.php?RecordID=459942.

## Guarantor

Chunsheng Wang, Jinmiao Chen, and Lai Wei.

## Data availability statement

We declare that all the raw data supporting the conclusions of this meta-analysis are available upon reasonable request.

## Provenance and peer review

We declare that our paper was not invited.

## Supplementary Material

SUPPLEMENTARY MATERIAL
