# Supplementary material for: Transcatheter tricuspid valve intervention versus medical therapy for symptomatic tricuspid regurgitation: a meta-analysis of reconstructed time-to-event data
Source: Int J Surg. 2024 Jun 13;110(10):6800–9. doi: 10.1097/JS9.0000000000001773 (PMC11487027; doi:10.1097/JS9.0000000000001773)
Supplement: SUPPLEMENTARY MATERIAL [file js9-110-6800-s001.docx]

**PubMed:**

(((Transcatheter OR Percutaneous OR Catheterization) AND (Edge-to-edge repair OR Repair OR Replacement OR Implantation OR Intervention)) OR MitraClip OR TriClip OR PASCAL OR FORMA OR Trialign OR TriCinch OR Cardioband OR TRAIPTA OR K-Clip OR Navigate OR EVOQUE OR LuX-Valve OR Cardiovalve) **AND** ((Medical therapy) OR (Therapy, Medical) OR (Medical therapies) OR (Therapies, Medical) OR (Medical treatment) OR (Treatment, Medical) OR (Medical treatments) OR (Treatments, medical) OR Medicine OR Medication OR Medications OR (Drug therapy) OR (Therapy, Drug) OR (Drug Therapies) OR (Therapies, Drug) OR Chemotherapy OR Chemotherapies OR Pharmacotherapy OR Pharmacotherapies OR (Conservative Treatment) OR (Conservative Treatments) OR (Treatment, Conservative) OR (Treatments, Conservative) OR (Conservative Management) OR (Conservative Managements) OR (Management, Conservative) OR (Managements, Conservative) OR (Conservative Therapy) OR (Conservative Therapies) OR (Therapies, Conservative) OR (Therapy, Conservative)) **AND** ((Tricuspid Valve Insufficiency[Title/Abstract]) OR (Insufficiency, Tricuspid Valve[Title/Abstract]) OR (Valve Insufficiency, Tricuspid[Title/Abstract]) OR (Tricuspid Valve Regurgitation[Title/Abstract]) OR (Regurgitation, Tricuspid Valve[Title/Abstract]) OR (Valve Regurgitation, Tricuspid[Title/Abstract]) OR (Tricuspid Valve Incompetence[Title/Abstract]) OR (Incompetence, Tricuspid Valve[Title/Abstract]) OR (Valve Incompetence, Tricuspid[Title/Abstract]) OR (Tricuspid Incompetence[Title/Abstract]) OR (Incompetence, Tricuspid[Title/Abstract]) OR (Tricuspid Regurgitation[Title/Abstract]) OR (Regurgitation, Tricuspid[Title/Abstract]) OR (Heart Valve Insufficiency, Tricuspid[Title/Abstract]) OR (Heart Valve Regurgitation, Tricuspid[Title/Abstract]) OR (Regurgitation, Right Atrioventricular Heart Valve[Title/Abstract]) OR (Right Atrioventricular Cardiac Regurgitation[Title/Abstract]) OR (Right Atrioventricular Cardiac Valve Insufficiency[Title/Abstract]) OR (Right Atrioventricular Cardiac Valvular Insufficiency[Title/Abstract]) OR (Right Atrioventricular Cardiac Valvular Regurgitation[Title/Abstract]) OR (Right Atrioventricular Heart Valve Insufficiency[Title/Abstract]) OR (Right Atrioventricular Heart Valve Regurgitation[Title/Abstract]) OR (Right Atrioventricular Heart Valvular Regurgitation[Title/Abstract]) OR (Right Atrioventricular Valve Insufficiency[Title/Abstract]) OR (Right Atrioventricular Valve Regurgitation[Title/Abstract]) OR (Right Atrioventricular Valvular Insufficiency[Title/Abstract]) OR (Right Atrioventricular Valvular Regurgitation[Title/Abstract]) OR (Tricuspid Cardiac Valve Insufficiency[Title/Abstract]) OR (Tricuspid Cardiac Valve Regurgitation[Title/Abstract]) OR (Tricuspid Cardiac Valvular Insufficiency[Title/Abstract]) OR (Tricuspid Cardiac Valvular Regurgitation[Title/Abstract]) OR (Tricuspid Heart Valve Insufficiency[Title/Abstract]) OR (Tricuspid Heart Valve Regurgitation[Title/Abstract]) OR (Tricuspid Heart Valvular Insufficiency[Title/Abstract]) OR (Tricuspid Heart Valvular Regurgitation[Title/Abstract]) OR (Tricuspid Insufficiency[Title/Abstract]) OR (Tricuspid Valvular Insufficiency[Title/Abstract]) OR (Tricuspid Valvular Regurgitation[Title/Abstract]) OR (Tricuspidal Insufficiency[Title/Abstract]))

**Embase:**

(((Transcatheter OR Percutaneous OR Catheterization) AND (‘Edge-to-edge repair’ OR Repair OR Replacement OR Implantation OR Intervention)) OR MitraClip OR TriClip OR PASCAL OR FORMA OR Trialign OR TriCinch OR Cardioband OR TRAIPTA OR ‘K-Clip’ OR Navigate OR EVOQUE OR ‘LuX-Valve’ OR Cardiovalve) **AND** (‘Medical therapy’ OR ‘Therapy, Medical’ OR ‘Medical therapies’ OR ‘Therapies, Medical’ OR ‘Medical treatment’ OR ‘Treatment, Medical’ OR ‘Medical treatments’ OR ‘Treatments, medical’ OR Medicine OR Medication OR Medications OR ‘Drug therapy’ OR ‘Therapy, Drug’ OR ‘Drug Therapies’ OR ‘Therapies, Drug’ OR Chemotherapy OR Chemotherapies OR Pharmacotherapy OR Pharmacotherapies OR ‘Conservative Treatment’ OR ‘Conservative Treatments’ OR ‘Treatment, Conservative’ OR ‘Treatments, Conservative’ OR ‘Conservative Management’ OR ‘Conservative Managements’ OR ‘Management, Conservative’ OR ‘Managements, Conservative’ OR ‘Conservative Therapy’ OR ‘Conservative Therapies’ OR ‘Therapies, Conservative’ OR ‘Therapy, Conservative’) **AND** ((‘Tricuspid Valve Insufficiency’:ab,ti) OR (‘Insufficiency, Tricuspid Valve’:ab,ti) OR (‘Valve Insufficiency, Tricuspid’:ab,ti) OR (‘Tricuspid Valve Regurgitation’:ab,ti) OR (‘Regurgitation, Tricuspid Valve’:ab,ti) OR (‘Valve Regurgitation, Tricuspid’:ab,ti) OR (‘Tricuspid Valve Incompetence’:ab,ti) OR (‘Incompetence, Tricuspid Valve’:ab,ti) OR (‘Valve Incompetence, Tricuspid’:ab,ti) OR (‘Tricuspid Incompetence’:ab,ti) OR (‘Incompetence, Tricuspid’:ab,ti) OR (‘Tricuspid Regurgitation’:ab,ti) OR (‘Regurgitation, Tricuspid’:ab,ti) OR (‘Heart Valve Insufficiency, Tricuspid’:ab,ti) OR (‘Heart Valve Regurgitation, Tricuspid’:ab,ti) OR (‘Regurgitation, Right Atrioventricular Heart Valve’:ab,ti) OR (‘Right Atrioventricular Cardiac Regurgitation’:ab,ti) OR (‘Right Atrioventricular Cardiac Valve Insufficiency’:ab,ti) OR (‘Right Atrioventricular Cardiac Valvular Insufficiency’:ab,ti) OR (‘Right Atrioventricular Cardiac Valvular Regurgitation’:ab,ti) OR (‘Right Atrioventricular Heart Valve Insufficiency’:ab,ti) OR (‘Right Atrioventricular Heart Valve Regurgitation’:ab,ti) OR (‘Right Atrioventricular Heart Valvular Regurgitation’:ab,ti) OR (‘Right Atrioventricular Valve Insufficiency’:ab,ti) OR (‘Right Atrioventricular Valve Regurgitation’:ab,ti) OR (‘Right Atrioventricular Valvular Insufficiency’:ab,ti) OR (‘Right Atrioventricular Valvular Regurgitation’:ab,ti) OR (‘Tricuspid Cardiac Valve Insufficiency’:ab,ti) OR (‘Tricuspid Cardiac Valve Regurgitation’:ab,ti) OR (‘Tricuspid Cardiac Valvular Insufficiency’:ab,ti) OR (‘Tricuspid Cardiac Valvular Regurgitation’:ab,ti) OR (‘Tricuspid Heart Valve Insufficiency’:ab,ti) OR (‘Tricuspid Heart Valve Regurgitation’:ab,ti) OR (‘Tricuspid Heart Valvular Insufficiency’:ab,ti) OR (‘Tricuspid Heart Valvular Regurgitation’:ab,ti) OR (‘Tricuspid Insufficiency’:ab,ti) OR (‘Tricuspid Valvular Insufficiency’:ab,ti) OR (‘Tricuspid Valvular Regurgitation’:ab,ti) OR (‘Tricuspidal Insufficiency’:ab,ti))

**Cochrane library:**

(((Transcatheter OR Percutaneous OR Catheterization) AND (Edge-to-edge repair OR Repair OR Replacement OR Implantation OR Intervention)) OR MitraClip OR TriClip OR PASCAL OR FORMA OR Trialign OR TriCinch OR Cardioband OR TRAIPTA OR K-Clip OR Navigate OR EVOQUE OR LuX-Valve OR Cardiovalve) **in All Text** **AND** ((Medical therapy) OR (Therapy, Medical) OR (Medical therapies) OR (Therapies, Medical) OR (Medical treatment) OR (Treatment, Medical) OR (Medical treatments) OR (Treatments, medical) OR Medicine OR Medication OR Medications OR (Drug therapy) OR (Therapy, Drug) OR (Drug Therapies) OR (Therapies, Drug) OR Chemotherapy OR Chemotherapies OR Pharmacotherapy OR Pharmacotherapies OR (Conservative Treatment) OR (Conservative Treatments) OR (Treatment, Conservative) OR (Treatments, Conservative) OR (Conservative Management) OR (Conservative Managements) OR (Management, Conservative) OR (Managements, Conservative) OR (Conservative Therapy) OR (Conservative Therapies) OR (Therapies, Conservative) OR (Therapy, Conservative)) **in All Text AND** (Tricuspid Valve Insufficiency) OR (Insufficiency, Tricuspid Valve) OR (Valve Insufficiency, Tricuspid) OR (Tricuspid Valve Regurgitation) OR (Regurgitation, Tricuspid Valve) OR (Valve Regurgitation, Tricuspid) OR (Tricuspid Valve Incompetence) OR (Incompetence, Tricuspid Valve) OR (Valve Incompetence, Tricuspid) OR (Tricuspid Incompetence) OR (Incompetence, Tricuspid) OR (Tricuspid Regurgitation) OR (Regurgitation, Tricuspid) OR (Heart Valve Insufficiency, Tricuspid) OR (Heart Valve Regurgitation, Tricuspid) OR (Regurgitation, Right Atrioventricular Heart Valve) OR (Right Atrioventricular Cardiac Regurgitation) OR (Right Atrioventricular Cardiac Valve Insufficiency) OR (Right Atrioventricular Cardiac Valvular Insufficiency) OR (Right Atrioventricular Cardiac Valvular Regurgitation) OR (Right Atrioventricular Heart Valve Insufficiency) OR (Right Atrioventricular Heart Valve Regurgitation) OR (Right Atrioventricular Heart Valvular Regurgitation) OR (Right Atrioventricular Valve Insufficiency) OR (Right Atrioventricular Valve Regurgitation) OR (Right Atrioventricular Valvular Insufficiency) OR (Right Atrioventricular Valvular Regurgitation) OR (Tricuspid Cardiac Valve Insufficiency) OR (Tricuspid Cardiac Valve Regurgitation) OR (Tricuspid Cardiac Valvular Insufficiency) OR (Tricuspid Cardiac Valvular Regurgitation) OR (Tricuspid Heart Valve Insufficiency) OR (Tricuspid Heart Valve Regurgitation) OR (Tricuspid Heart Valvular Insufficiency) OR (Tricuspid Heart Valvular Regurgitation) OR (Tricuspid Insufficiency) OR (Tricuspid Valvular Insufficiency) OR (Tricuspid Valvular Regurgitation) OR (Tricuspidal Insufficiency) **in Title Abstract Keyword**
