# Supplementary figures and images for: Transcatheter tricuspid valve intervention versus medical therapy for symptomatic tricuspid regurgitation: a meta-analysis of reconstructed time-to-event data
Source: Int J Surg. 2024 Jun 13;110(10):6800–9. doi: 10.1097/JS9.0000000000001773 (PMC11487027; doi:10.1097/JS9.0000000000001773)

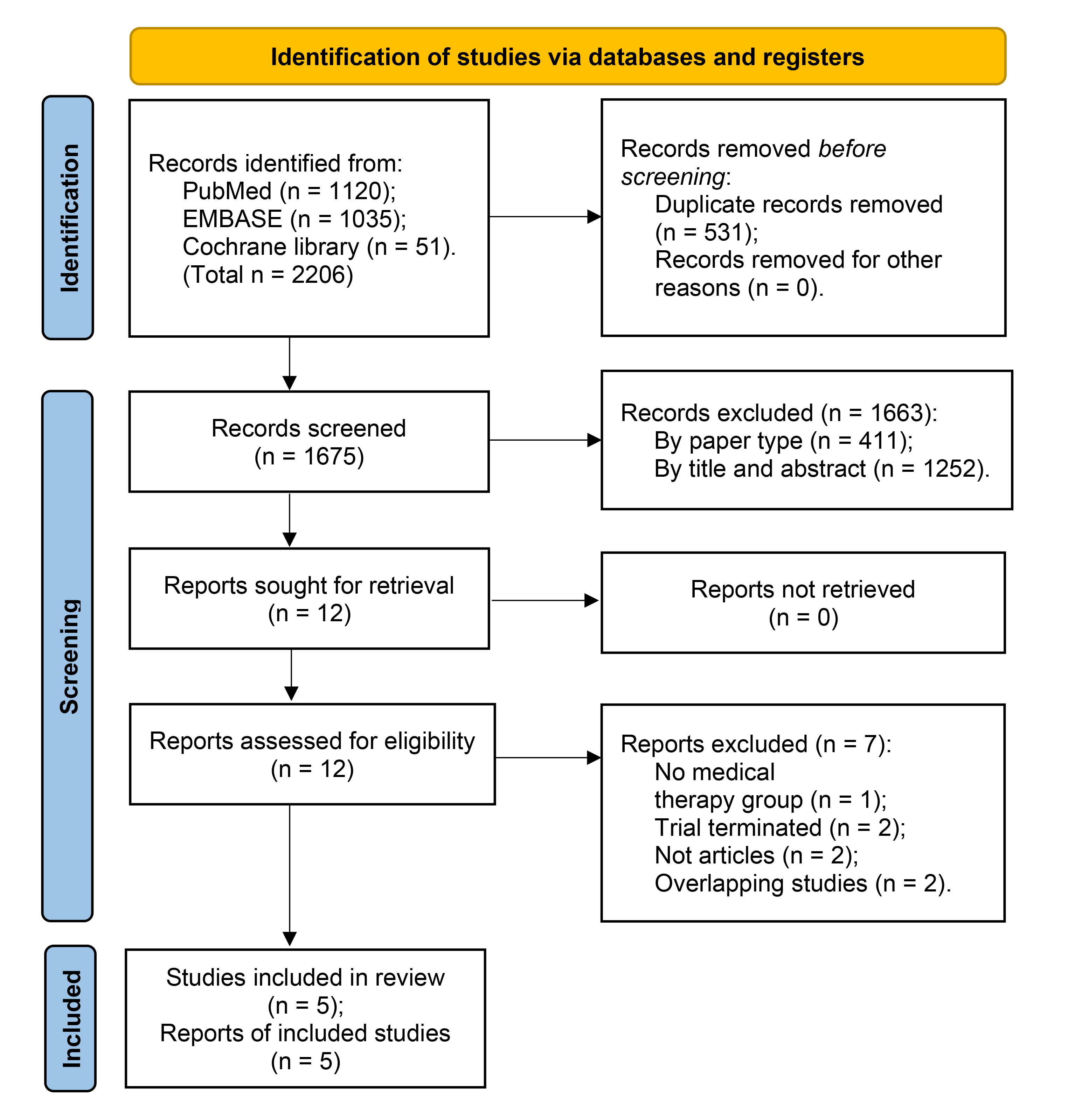

Supplement: SUPPLEMENTARY MATERIAL [file js9-110-6800-s003.tif]
