# Supplementary material for: Transcatheter tricuspid valve intervention versus medical therapy for symptomatic tricuspid regurgitation: a meta-analysis of reconstructed time-to-event data
Source: Int J Surg. 2024 Jun 13;110(10):6800–9. doi: 10.1097/JS9.0000000000001773 (PMC11487027; doi:10.1097/JS9.0000000000001773)
Supplement: SUPPLEMENTARY MATERIAL [file js9-110-6800-s005.docx]

**
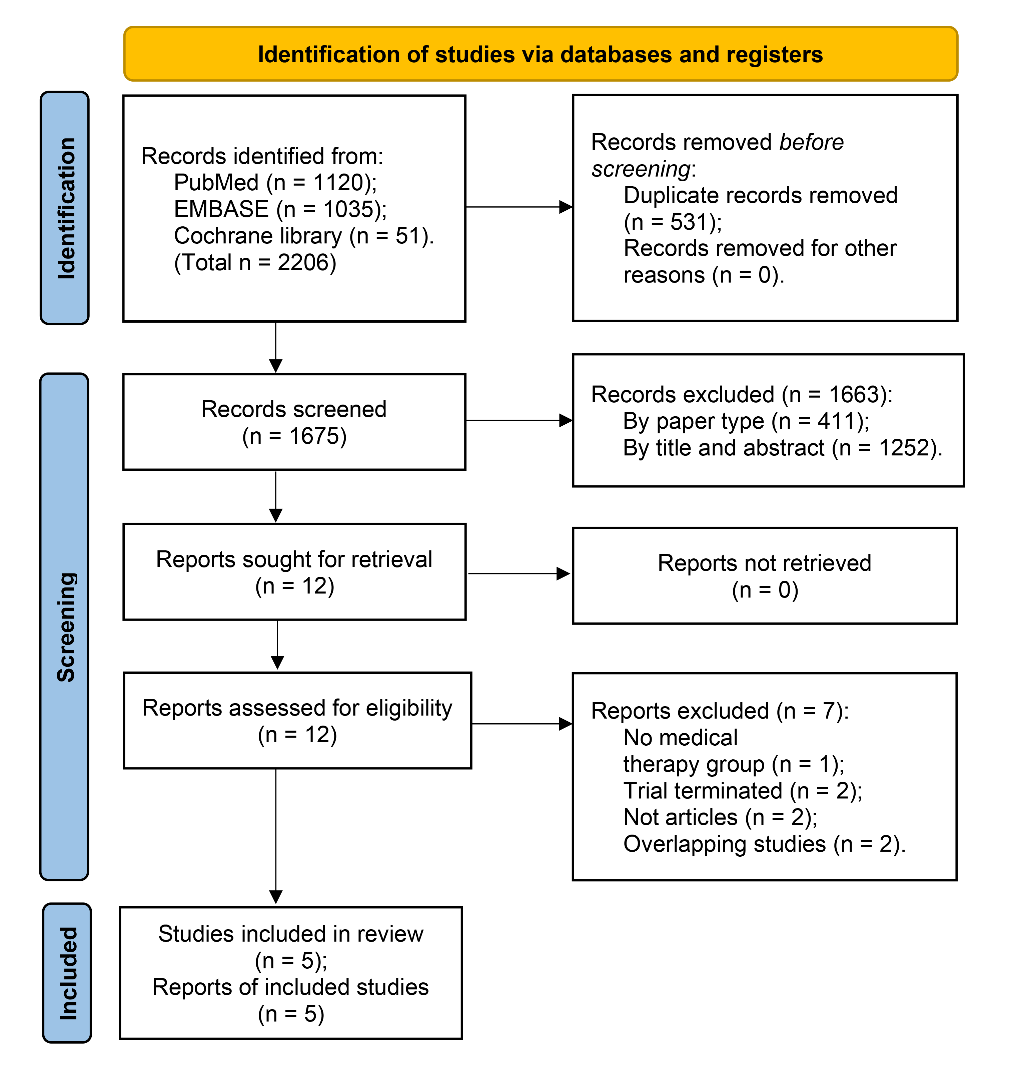
**

**Supplementary Figure S1**. PRISMA flow diagram for literature search and inclusion.

**
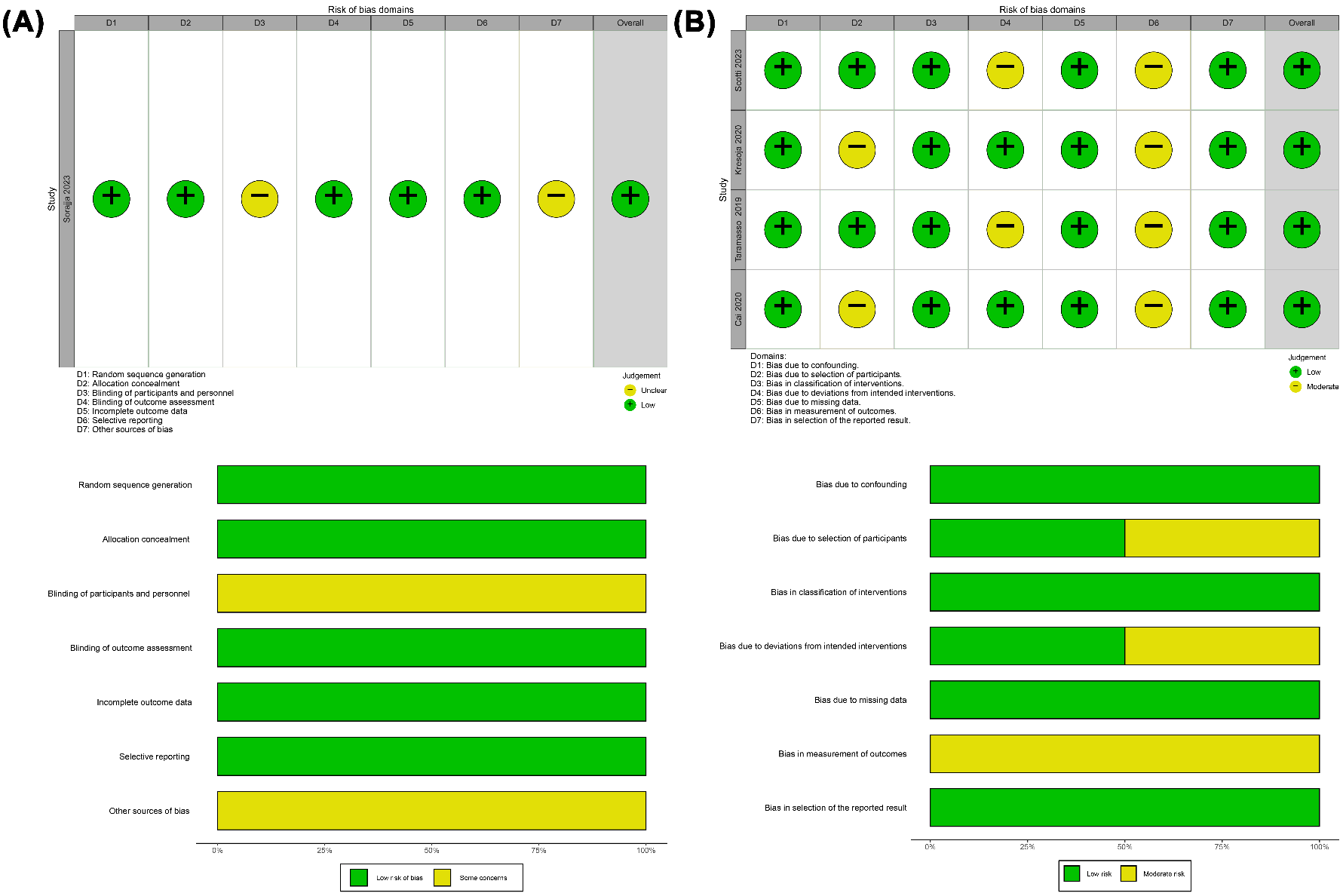
**

**Supplementary Figure S2**. Quality assessment plots of (A) the included randomized controlled trial based on the Cochrane Risk of Bias 2 tool and (B) the included observational studies based on the Cochrane Risk of Bias in Non-Randomized Studies of Interventions tool.

**
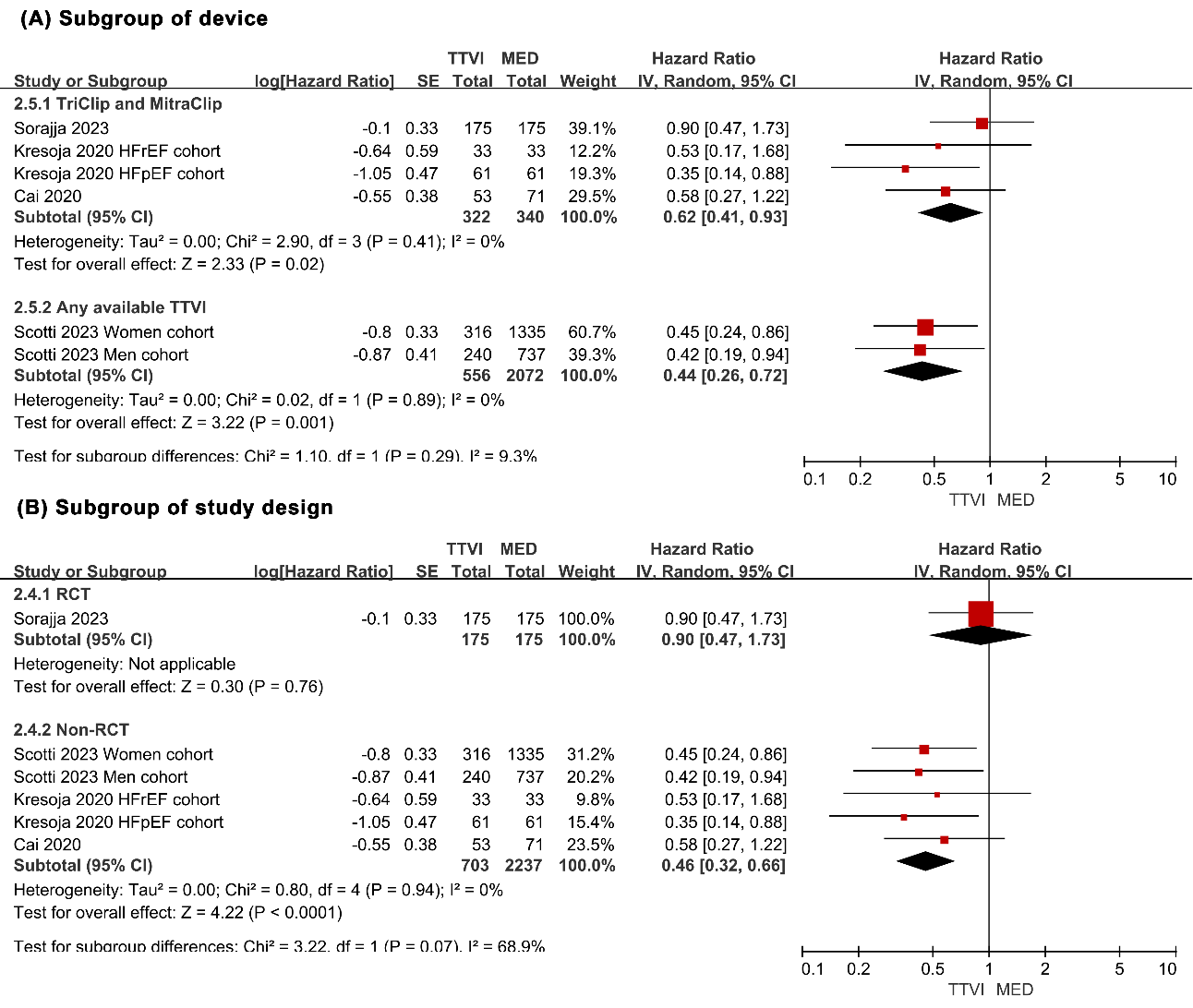
**

**Supplementary Figure S3**. Forest plots of subgroup analyses showing all-cause mortality for hazard ratio. (A) Analyzed by the subgroup of device type. (B) Analyzed by the subgroup of study design. CI, confidence interval; IV, inverse-variance; MED, medical therapy; TTVI, transcatheter tricuspid valve intervention.





**Supplementary Figure S4**. Funnel plots showing the publication bias. (A) All-cause mortality. (B) Heart failure hospitalization. (C) The composite outcome of all-cause mortality and heart failure hospitalization. SE, standard error.


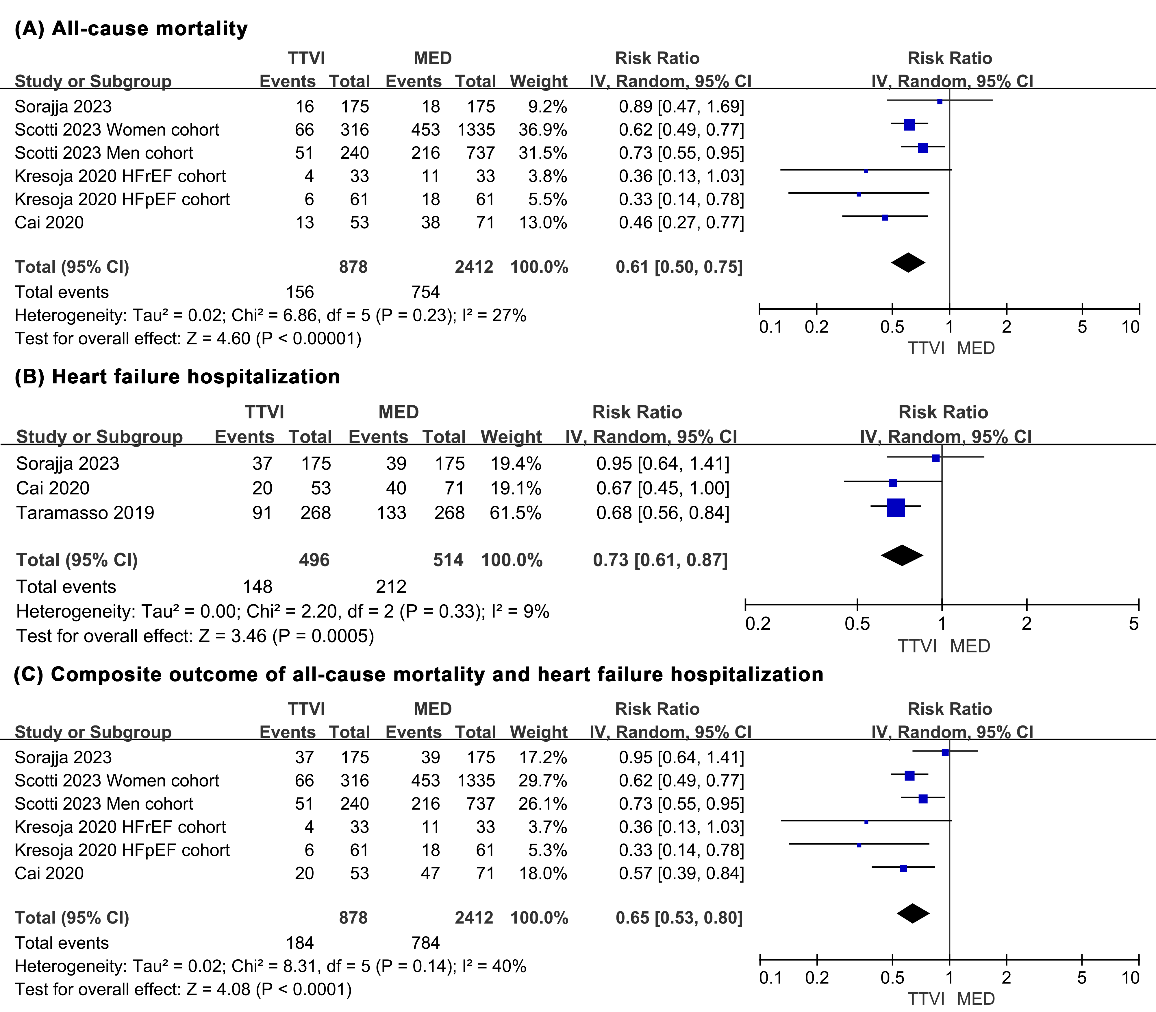


**Supplementary Figure S5**. Forest plots showing the pooled 1-year outcomes for risk ratio. (A) All-cause mortality. (B) Heart failure hospitalization. (C) The composite outcome of all-cause mortality and heart failure hospitalization. CI, confidence interval; IV, inverse-variance; MED, medical therapy; TTVI, transcatheter tricuspid valve intervention.

**Table S1**. Results of publication bias assessment in different clinical outcomes.

| Outcomes | Number of  included studies | Publication bias (*P* value) | | |
| --- | --- | --- | --- | --- |
|  |  | Egger’s test | Begg’s test | arcsine-Thompson’s test |
| All-cause mortality | 4 | 0.45 | 0.45 | 0.49 |
| Heart failure hospitalization | 3 | 0.44 | 0.30 | 0.52 |
| Composite outcome | 4 | 0.36 | 0.71 | 0.38 |

**Table S2**. Leave-one-out analysis for all-cause mortality.

| Excluded study | TTVI versus MED | | |
| --- | --- | --- | --- |
|  | HR (95% CI) | Heterogeneity | |
|  |  | *I*^2^, % | *P* value |
| None | 0.54 (0.39–0.74) | 0 | 0.55 |
| Sorajja 2023 | 0.46 (0.32–0.66) | 0 | 0.94 |
| Scotti 2023 Women cohort | 0.57 (0.40–0.82) | 0 | 0.46 |
| Scotti 2023 Men cohort | 0.56 (0.40–0.80) | 0 | 0.47 |
| Kresoja 2020 HFrEF cohort | 0.54 (0.39–0.75) | 1 | 0.40 |
| Kresoja 2020 HFpEF cohort | 0.57 (0.41–0.80) | 0 | 0.55 |
| Cai 2020 | 0.53 (0.38–0.75) | 0 | 0.41 |

CI, confidence interval; HFpEF, heart failure with preserved ejection fraction; HFrEF, heart failure with reduced ejection fraction; MED, medical therapy; HR, hazard ratio; TTVI, transcatheter tricuspid valve intervention.
